# Idiopathic Premature Ventricular Contractions From the Outflow Tract Display an Underlying Substrate That Can Be Unmasked by a Type 2 Brugada Electrocardiographic Pattern at High Right Precordial Leads

**DOI:** 10.3389/fphys.2020.00969

**Published:** 2020-08-07

**Authors:** Leonor Parreira, Rita Marinheiro, Pedro Carmo, Dinis Mesquita, José Farinha, Pedro Amador, António Ferreira, Marta Fonseca, Francisco Costa, Diogo Cavaco, Rui Caria, Pedro Adragão

**Affiliations:** ^1^Department of Cardiology, Hospital da Luz Lisboa, Lisbon, Portugal; ^2^Department of Cardiology, Hospital Center of Setúbal, Setúbal, Portugal

**Keywords:** idiopathic arrhythmias, right ventricular outflow tract, low voltage, Brugada pattern, catheter ablation

## Abstract

**Background:** Patients with premature ventricular contractions (PVCs) from the right ventricular outflow tract (RVOT) and apparently normal hearts, can have ST elevation similar to type 2 or type 3 Brugada pattern in the electrocardiographic (ECG) performed at a higher position. Cardiac magnetic resonance (CMR), has shown conflicting data regarding existence of structural abnormalities in patients with idiopathic PVCs from the RVOT.

**Objective:** Our aim was to evaluate the prevalence of low voltage areas (LVAs) in the RVOT of patients with PVCS from the outflow tract, and in a control group. Secondly, assess for the presence of a non-invasive ECG marker.

**Methods:** A 56 consecutive patients, 45 with frequent PVCs (>10000/24 h) LBBB, vertical axis, negative in aVL and 11 subjects without PVCs. Arrhythmogenic right ventricular cardiomyopathy was ruled out in all patients. An ECG was performed with V1–V2 at the level of the second intercostal space and the presence of ST-segment elevation with a Type 2 or 3 Brugada pattern (Type 2 BrP) was assessed. Bipolar voltage map of the RVOT was performed in sinus rhythm (0.5–1.5 mV color display). Areas with electrograms <1.5 mV represented the LVA. The area adjacent to the pulmonary valve usually displays voltage between 0.5 and 1.5 mV and is classified as transitional-voltage zone. Presence of LVAs outside this transitional-voltage zone were estimated. We compared two groups with and without ST-segment elevation and tested for the association between ECG pattern and LVAs.

**Results:** None of the patients in the control group had ST-segment elevation or LVAs. In the PVC group, no patient had type 1 Brugada pattern, 29 patients (64%) had type 2 or 3 ST-segment elevation (Type 2 BrP), and 28 (62%) had LVAs outside the transitional-voltage zone. LVAs were more frequent in patients with Type 2 BrP; 93% versus 4%, *p* < 0.0001. The ECG pattern was associated with the presence of LVAs, OR (95% CI): 202.50 (16.92–2423), *p* < 0.0001.

**Conclusion:** Low voltage areas were frequently present in the RVOT of patients with idiopathic PVCs. They were absent in controls and can be unmasked by the presence of Type 2 BrP in high right precordial leads.

## Introduction

Idiopathic premature ventricular contractions (PVCs) arise from the outflow tracts in more than 80% of cases, more frequently the right ventricular outflow tract (RVOT) (Lerman, 2015). Cardiac magnetic resonance (CMR) imaging studies have shown conflicting data regarding the existence of structural abnormalities in the RVOT of those patients. Initial studies documented the presence of localized wall bulging, focal wall thinning or fatty replacement in a high percentage of patients (Globits et al., 1997). However, most recent studies using electrocardiographic (ECG) gating and imaging with late gadolinium enhancement (LGE) have shown absence of pathological findings in patients with idiopathic RVOT PVCs (Markowitz et al., 2014).

Detection of myocardial fibrosis can be assessed non-invasively with CMR using LGE (Bing and Dweck, 2019) but its detection depends on the type of fibrosis, whether replacement or interstitial fibrosis. In the initial phases of non-ischemic cardiomyopathy for instance, although a certain degree of diffuse fibrosis may be present, it goes undetected by LGE techniques and may be detected by T1 mapping (Puntmann et al., 2016).

Previous studies have shown presence of low voltage areas (LVAs) in the RVOT of patients undergoing catheter ablation of frequent PVCs despite normal CMR (Yamashina et al., 2009; Furushima et al., 2012; Letsas et al., 2019; Parreira et al., 2019a, b). These findings may suggest the presence of an underlying substrate too subtle to be identified by CMR techniques (Santangeli et al., 2011). The Brugada syndrome, caused by an inherited sodium channelopathy, is diagnosed in patients with Type 1 ST elevation, spontaneous or after drug provocation, at the standard or high position, and in patients with baseline Type 2 pattern that converts to Type 1 with drug provocation (Antzelevitch et al., 2005, 2017; Bayés de Luna et al., 2012; Priori et al., 2015). Patients with PVCs from the RVOT and apparently normal hearts can have ST elevation at V1 obtained at the level of the second intercostal space (ICS) similar to type 2 or type 3 Brugada patterns (Parreira et al., 2019b). That ECG pattern was associated with the presence of LVAs in the RVOT.

The aim of this study was to evaluate the prevalence of both the ST-segment elevation at high right ventricular leads and that of LVAs in the RVOT, in idiopathic patients with frequent PVCS from the outflow tracts and in a control group. Secondly, estimate the value of ST-segment elevation as a non-invasive ECG marker of low voltage in the RVOT.

## Materials and Methods

### Patient Population

From 2016 to 2020, we retrospectively studied consecutive patients with symptomatic idiopathic frequent PVCs (>10000/24 h) with a LBBB, vertical axis, negative in aVL that were referred for catheter ablation by the same operator. Patients that did not undergo electroanatomical voltage map of the RVOT in sinus rhythm were excluded. The study was carried out in two hospitals. All patients underwent transthoracic echocardiography, including 2-dimensional, M-mode, and Doppler study and standard 12-lead ECG. A second ECG was obtained with the right ventricular leads at the level of the second ICS. A treadmill exercise test was performed if symptoms appeared or were aggravated by exercise. All patients with PVCs had a CMR with Gadolinium to exclude the presence of RVOT anomalies.

Arrhythmogenic right ventricular cardiomyopathy was ruled out according to the Task Force Criteria (Marcus et al., 2010). A 24-h Holter recording was performed before ablation and the number of PVCs per 24 h and the presence of episodes of non-sustained ventricular tachycardia (NSVT), defined as >3 PVCs in a run were assessed. Patients with evidence of conduction delays, electrical diseases or abnormal QRS morphology, as well as patients with previous ablation were excluded.

A control group of consecutive patients without PVCs, that underwent catheter ablation of supraventricular tachycardias since 2019 and agreed to have a voltage map of the RVOT performed in sinus rhythm was also studied.

### Study Design

We retrospectively assessed the presence of ST-segment elevation at the level of the second ICS, in patients with PVCs and in controls. According to the J-Wave syndromes expert consensus conference report (Antzelevitch et al., 2017) three different types of ST-segment elevation described as Brugada-type ECG patterns, may be observed during ECG recording: type 1 has a coved ST segment elevation ≥2 mm, negative T wave and no isoelectric separation of T wave; type-2 has a saddleback appearance with an ST segment elevation of ≥2 mm, a trough displaying >1 mm ST elevation and then either a positive or biphasic T wave; type 3 has either a saddleback or coved appearance with an ST-segment elevation of <1 mm. Type 2 and type 3 ECG are not diagnostic of the Brugada syndrome. The ST-segment elevation observed in our patients was classified according to the above classification. Patients were divided in two groups according to the presence of an ST-segment elevation similar to any of the patterns described above. Both groups with and without ST-segment elevation were compared regarding demographic and clinical characteristics, echocardiographic ECG and 24 Holter data and electroanatomical mapping and ablation data. The association between the presence of ST-segment elevation and presence of LVAs was analyzed.

### Standard 12-Leads ECG and High Right Precordial Leads ECG

The ECG was performed with standard paper speed and calibration. After a standard 12-lead ECG recording the ECG was repeated, with V1 and V2 leads placed in the second ICS and maintaining the other lead’s position. The duration of the QRS in sinus rhythm and the precordial transition of the sinus and ectopic beats, defined as the precordial lead where the QRS changes from predominately negative to predominately positive and the R/S ratio becomes >1 were assessed in the standard ECG both in sinus rhythm and during the PVC. The presence of T wave inversion beyond V1 was evaluated in standard and high right precordial leads ECG.

The ST-segment elevation was measured at the take-off point of the QRS-ST and the morphology of the ST segment was analyzed. All ECG recordings were evaluated by two independent reviewers blinded to the result of the voltage map.

### Electroanatomic Mapping and Ablation

All patients underwent electroanatomical mapping with CARTO 3 (Biosense Webster) or EnSite Velocity (Abbott). With the former, all procedures were performed using the Niobe magnetic navigation system (Stereotaxis) working with the monoplane fluoroscopy system AXIOM Artis (Siemens) as previously described (Parreira et al., 2013). An irrigated tip Navistar RMT Thermocool catheter (Biosense Webster) was used with a 3.5-mm distal tip electrode and a 2-5-2 interelectrode distance. With the EnSite Velocity system all procedures were done manually with the monoplane fluoroscopy system BV Pulsera (Philips) and using an irrigated tip Therapy Cool Path or FlexAbility catheter (Abbott) with a 4-mm distal tip electrode and a 1-4-1 interelectrode distance. The ablation catheter was introduced via the femoral vein, manually advanced to the right atrium and then automatically advanced to the His bundle and RVOT in the magnetic navigation system patients or manually in the EnSite patients, under fluoroscopic guidance. The ablation catheter was then placed at multiple sites on the endocardial surface of the RVOT. The 12-lead surface ECGs and intracardiac electrograms were recorded simultaneously by a digital multichannel system, filtered at 30–300 Hz for bipolar electrograms and at 0.05–525 Hz for unipolar electrograms, displayed at 100 mm/s speed. Two maps were created, a voltage bipolar map in sinus rhythm and an activation map during the PVC. In sinus rhythm the electrograms were analyzed in regard of their amplitude and the information was used to generate a 3-dimensional electroanatomical voltage map of the RVOT, with the electrophysiologic information, color-coded and superimposed on the geometry. The color display for voltage mapping ranged from purple, representing electroanatomical normal tissue (amplitude ≥1.5 mV), to red, representing electroanatomical scar tissue (amplitude <0.5 mV). LVAs were defined as areas with bipolar electrograms with an amplitude <1.5 mV. The level of RVOT/pulmonary valve junction was thoroughly determined based on electroanatomical voltage mapping by passing the catheter into the pulmonary artery and slowly withdrawing it to the RVOT. The voltage above the pulmonary valve is usually less than 0.5 mV. The area immediately below the level of the pulmonary valve displays intermediate colors, corresponding to a bipolar voltage between 0.5 and 1.5 mV, defined as the transitional-voltage zone (Yamashina et al., 2009). Presence of LVAs outside the transitional-voltage zone, were assessed.

The activation map was created by mapping several points during each PVCs while using a surface ECG lead as reference. The ablation site was selected based on the earliest endocardial activation time with a QS pattern at the unipolar electrogram and confirmed by the pace mapping that provided at least 11 out of 12 pace matches between paced and spontaneous PVCs. Energy was delivered from an EP Shuttle RF generator (Stockert) between the distal electrode of the ablation catheter and a cutaneous patch, for up to 120 s, to a maximum temperature of 43°C and a power output limit of 50 W. When the application was ineffective, additional applications were delivered to sites adjacent to the earliest activation site. During ablation, light sedation with midazolam (bolus) or remifentanil (continuous perfusion) was administered when needed. Success was defined as abolition of PVCs under isoprenaline infusion until 30 min after ablation. All the intracardiac electrograms were reviewed by two senior electrophysiologists blinded to the results of the ECG.

### Statistical Analysis

All analyses were performed using SPSS statistical software, version 25.0 (SPSS, Inc., Chicago, IL, United States). Data is presented as median and lower and upper quartile (Q_1_–Q_3_) for continuous variables and as absolute numbers and percentages for categorical variables. Continuous variables were compared with the use of Mann Whitney test for independent samples. Categorical variables were compared with the use of two-side Fischer’s exact-test or the chi square test as appropriate for independent samples and with the McNemar test for related samples. Univariable logistic regression analysis and calculation of the respective odds ratios (OR) and 95% confidence intervals (CI) was used to evaluate the discriminative power of ST-segment elevation as a marker of LVA in the RVOT. The performance of ST-segment elevation as a diagnostic test including the positive and negative predictive value as well as specificity and sensitivity was based on 2 × 2 contingency table and chi square test. For all tests a *p* value <0.05 was considered as statistically significant.

### Ethics

All patients signed the informed consent form and the study was approved by the Ethical Committee of both hospitals. The study is in compliance with the Helsinki Declaration.

## Results

### Patient Population

Fifty six patients were enrolled, 45 patients with PVCs and 11 patients in the control group of whom eight underwent ablation of atrioventricular nodal reentrant tachycardia, two of accessory pathways and one of typical atrial flutter. Both groups did not differ in relation to age or gender (Table 1). Patients in the PVC group were more frequently on beta blocker therapy, 73% versus 9%, *p* < 0.0001.

**TABLE 1:** Baseline characteristics and comparison between PVC group and control group.

	**Overall sample (*n* = 56)**	**PVC group (*n* = 45)**	**Control (*n* = 11)**	***p* value**
**Demographic data**				
Age in years, median (Q_1_–Q_3_)	50 (36–60)	48 (37–61)	50 (33–54)	0.773
Male Gender, *n* (%)	23 (41)	20 (44)	3 (27)	0.496
**Risk factors, history and medications**				
Hypertension, *n* (%)	7 (13)	6 (13)	1 (9)	1.000
Diabetes, *n* (%)	3 (5)	2 (4)	1 (9)	0.488
Syncope	5 (9)	5 (11)	0 (0)	0.571
Family history of sudden death	2 (4)	2 (5)	0 (0)	1.000
Beta blockers, *n* (%)	34 (61)	33 (73)	1 (9)	<0.0001
**Standard 12 lead ECG**				
QRS duration in ms, median (Q1–Q3)	82 (80–90)	84 (80–90)	80 (79–82)	0.062
T wave inversion beyond V1, *n* (%)	3 (5)	3 (7)	0 (0)	1.000
Type 2 BrP, *n* (%)	0 (0)	–	–	–
**High right precordial leads ECG**				
T wave inversion beyond V1, *n* (%)	18 (32)	18 (40)	0 (0)	0.011
Type 2 BrP, *n* (%)	29 (52)	29 (64)	0 (0)	<0.0001
24-h Holter Monitoring*				
Number of PVCs, median (Q1–Q3)*	–	20000 (14000–24000)	–	–
NSVT, *n* (%)*	–	10 (22)	–	–
**Echocardiogram**				
LVEF in %, median (Q_1_–Q_3_)	57 (56–60)	58 (56–60)	58 (57–59)	0.630

*In the PVC group. BrP, Brugada electrocardiographic pattern; LVEF, left ventricular ejection fraction; NSVT, non-sustained ventricular tachycardia; PVC, premature ventricular contractions.

In the PVC group, all patients were symptomatic, 44 complained of palpitations, one patient had episodes of dizziness and five patients had a history of fainting, all typically vagal in nature. Two patients had family history of sudden death in one due to a myocarditis and in the other at the age of 64 years and preceded by chest pain. Physical examination, and transthoracic echocardiography, including 2-dimensional, M-mode, and Doppler echocardiography were normal and demonstrated normal right ventricle size and function. The CMR did not show evidence of RVOT abnormalities in any patient.

Twenty patients underwent treadmill exercise test and twelve (60%) had a reduction of PVC frequency with exercise. The 24-h Holter recording showed a high PVC burden with a median of 20000 (14000–24000)/24 h and NSVT in 10 patients (22%).

### Standard 12 Lead ECG and High Right Precordial Leads ECG

The mean duration of the QRS was 82 (80–90) ms and three patients in the PVC group displayed T wave inversion beyond V1, not significantly different between the PVC and control group (Table 1).

**FIGURE 1:**
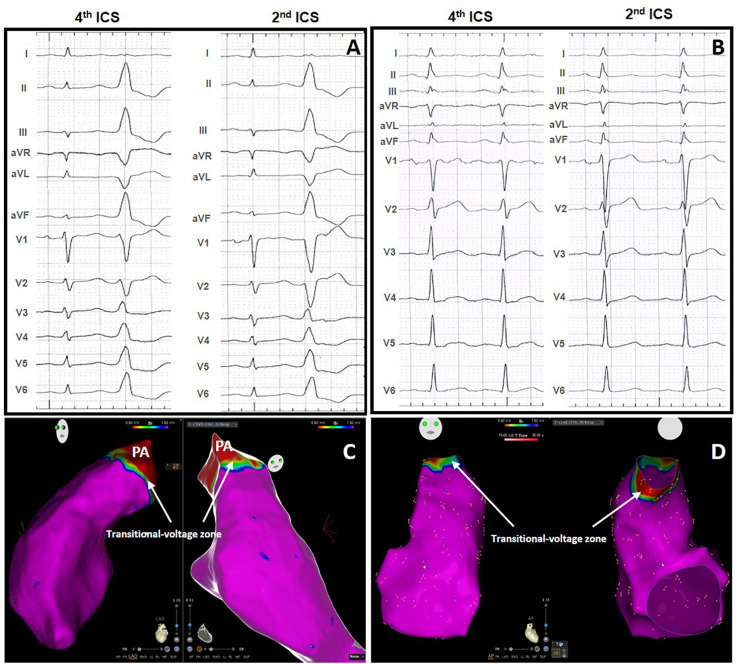
Standard 12-lead electrocardiographic (ECG) and high right ventricular leads ECG without ST elevation in a patient with PVCs **(A)** and in a control subject without PVCs **(B)**. Normal voltage map without LVAs in the patient with PVCs **(C)** and in the control subject **(D)**. PA, pulmonary artery. Indicating the transitional-voltage zone (white arrows).

Type 2 BrP ST-segment elevation was absent in the standard ECG in both groups. Eighteen patients displayed T wave inversion beyond V1 in the high ECG, which represents a six-fold increase in comparison with the standard ECG (*p* < 0.0001). No patient in the control group showed T wave inversion beyond V1 (Figure 1B).

**FIGURE 2:**
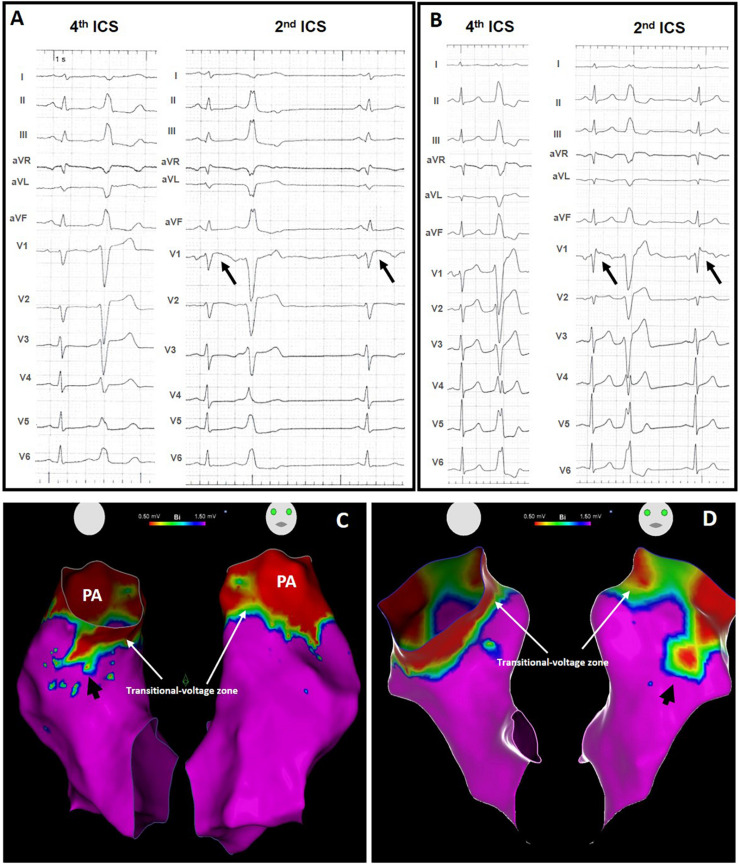
Standard 12-lead ECG and high right ventricular leads ECG with Type 2 BrP (black arrows) in two patients with PVCs from the RVOT **(A,B)** and respective voltage map showing an area of low voltage outside the transitional-voltage zone (white arrows), in the septal wall [**(C)**, black arrow] and in the free wall [**(D)**, black arrow]. PA, pulmonary artery.

An ST-segment elevation was present in V1 recorded at the second ICS, in 29 patients (64%) in the PVC group and was absent in the control group, *p* < 0.0001. The ST-segment elevation was coved-type but <1 mm in 27 patients (example in Figure 2A) and classified as type 3, and ≥2 mm but without the coved-type morphology in two (example in Figure 2B), classified as type 2. No definite type 1 pattern was observed. Types 2 and 3 were classified together as Type 2 BrP.

### Comparison Between Patients With and Without Type 2 BrP in High Right Precordial Leads ECG

The characteristics of patients with and without Type 2 BrP are depicted in Table 2. There were no significant differences regarding demographic data, clinical variables, PVC burden or presence of NSVT or standard ECG measurements. However, on the ECG performed in the high position, patients with Type 2 BrP showed T wave inversion beyond V1 more frequently (45% versus 19%, *p* = 0.047).

**TABLE 2:** Baseline characteristics of patients with and Type 2 BrP in high right precordial leads.

	**Overall sample (*n* = 56)**	**With Type 2 BrP (*n* = 29)**	**Without Type 2 BrP (*n* = 27)**	***p* value**
**Demographic data**				
Age in years, median (Q_1_–Q_3_)	50 (36–60)	45 (35–60)	50 (36–60)	0.896
Male Gender, *n* (%)	23 (41)	13 (45)	10 (37)	0.596
Patients with frequent PVCs, *n* (%)	45 (80)	29 (100)	16 (60)	<0.0001
**Risk factors, history and medications**				
Hypertension, *n* (%)	7 (13)	3 (10)	4 (14)	0.700
Diabetes, *n* (%)	3 (5)	1 (3)	2 (7)	0.605
Syncope, *n* (%)	5 (9)	1 (3)	4 (14)	0.185
Family history of sudden death, *n* (%)	3 (5)	0 (0)	3 (11)	0.106
Beta blockers, *n* (%)	34 (61)	21 (72)	13 (48)	0.100
24-h Holter Monitoring*				
Number of PVCs, median (Q1–Q3)*	20000 (14000–24000)	17595 (12774–24000)	20112 (15500–28500)	0.325
NSVT, *n* (%)*	10 (22)	6 (21)	4 (25)	0.726
**Standard 12 lead ECG**				
QRS duration in ms, median (Q1–Q3)	82 (80–90)	85 (80–90)	80 (80–90)	0.829
PVC precordial transition beyond V3, *n* (%)*	30 (68)	20 (69)	10 (63)	0.746
PVC transition earlier than SR, *n* (%)*	14 (31)	7 (24)	7 (44)	0.197
T wave inversion beyond V1, *n* (%)	3 (5)	3 (10)	0 (0)	0.237
**High right precordial leads ECG**				
T wave inversion beyond V1, *n* (%)	18 (32)	13 (45)	5 (19)	0.047
**Echocardiogram**				
LVEF in %, median (Q1–Q3)	57 (56–60)	57 (56–60)	60 (56–64)	0.254

*In the PVC group. BrP, Brugada electrocardiographic pattern; LVEF, left ventricular ejection fraction, NSVT, non-sustained ventricular tachycardia; PVC, premature ventricular contractions.

### Electroanatomical Mapping and Ablation

#### PVC Group Versus Control Group

The electroanatomical mapping was successfully acquired in all patients, the median number of points per patient, collected in the RVOT to obtain the voltage map was 142 (98–300) and the results are displayed in Table 3. The number of points sampled was not significantly different between patients with PVCs and the control group, respectively 141 (102–300) versus 182 (120–317), *p* = 0.529. The electroanatomical system used in control group was predominantly Carto (90%), while in the PVC group it was used in approximately 50% of cases, *p* = 0.036. This occurred because Carto was the system used with Stereotaxis, our choice for mapping the RVOT in the control group due to safety issues.

**TABLE 3:** Electroanatomical mapping and ablation data.

	**Overall sample (*n* = 56)**	**PVC group (*n* = 45)**	**Control (*n* = 11)**	***p* value**
**Electroanatomical mapping**				
Number of points in the map, median (Q_1_–Q_3_)	142 (98–300)	141 (102–300)	182 (120–317)	0.529
Carto/EnSite	34/22	24/21	10/1	0.036
LVAs, *n* (%)	28 (50)	28 (62)	0 (0)	<0.0001
Electroanatomical mapping and ablation*	**Overall PVC group (*n* = 45)**	**With Type 2 BrP (*n* = 29)**	**Without Type 2 BrP (*n* = 16)**	***p* value**
Number of points in the map, median ((Q_1_–Q_3_)*	141 (102–300)	152 (104–313)	118 (99–190)	0.066
Carto/EnSite*	24/21	13/16	11/5	0.212
PVCs from RVOT/LVOT*	40/5	26/3	14/2	1.000
LVAs, *n* (%)*	28 (62)	27 (93)	1 (4)	<0.0001
SOO in LVAs, *n* (%)*	18 (40)	17 (59)	1 (4)	0.001
Success, *n* (%)*	40 (89)	27 (93)	13 (81)	0.330

*In the PVC group. BrP, Brugada electrocardiographic pattern; LVOT, left ventricular outflow tract; LVA, low voltage area; RVOT, right ventricular outflow tract; SOO, site of origin.

In 40 patients the PVCs originated in the RVOT and in five the origin was in the left aortic cusp. Presence of LVAs outside the transitional-voltage zone were absent in all subjects from the control group (Figure 1) and present in 28 patients (62%) of the PVC group, *p* < 0.0001 (Figure 2).

#### Type 2 BrP as Risk Marker of Low Voltage Areas in Patients With PVCs

**FIGURE 3:**
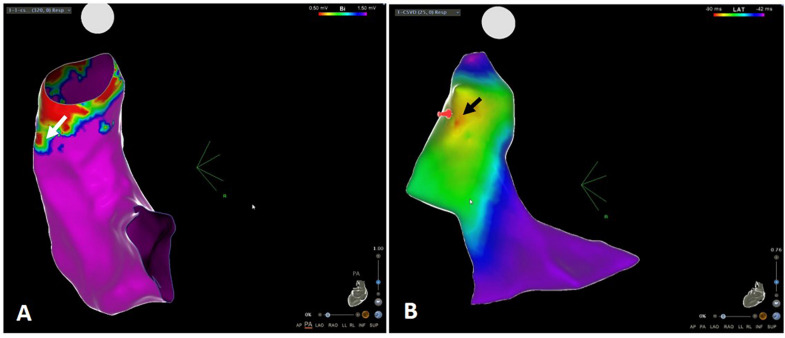
**(A)** Voltage map of the RVOT displaying low voltage area (white arrow). **(B)** Activation map of the same patient showing site of origin of the PVCs (black arrow) in the area of low voltage. RVOT, right ventricular outflow tract.

The number of points sampled for the RVOT map in the PVC group was 141 (102–300) not significantly different between patients with and without Type 2 BrP, respectively, 152 (104–313) versus 118 (99–190), *p* = 0.066 (Table 3). The electroanatomical system used was not significantly different in the two groups neither was the site of origin of the PVCs right versus left. Presence of LVAs outside the transitional-voltage zone were more frequent in the group with Type 2 BrP, 93% of cases versus 4%, *p* < 0.0001. The site of origin of the PVCs was in the LVA outside the transitional-voltage zone in 18 out of the 45 patients with PVCs (40%) (Figure 3). This percentage was significantly higher in patients with Type 2 BrP, respectively 59% of cases versus 4%, *p* = 0.001 (Table 3). The success rate was not significantly different in both groups. Type 2 BrP was a predictor of the presence of LVAs outside the transitional-voltage zone in the RVOT of patients with idiopathic PVCs, OR (95% CI) 202.50 (16.92–2423), *p* < 0.0001. The positive predictive value was 93%, negative predictive value 94%, sensitivity 96%, and specificity 88%.

## Discussion

The first finding of this study was the presence of a Type 2 BrP in 64% of patients with PVCs, on the ECG performed at the level of the second ICS. According to the contemporary definition of Brugada syndrome, the diagnostic ECG displays a coved-type ST segment elevation of at least 2 mm in one or more leads among the right precordial leads V1 and/or V2 positioned in the fourth, third or second ICS (Priori et al., 2015; Antzelevitch et al., 2017). The reason for using these higher positioned V1–V2 in the diagnosis of Brugada syndrome, is their closer proximity to the RVOT, now known to be the origin of the disease (Brugada et al., 2018). That is the reason why we used these higher leads in the present study, to record the electric activity form the RVOT. None of our patients, had a type 1 Brugada pattern (Antzelevitch et al., 2017). We did not perform drug challenge in our patients and that is in accordance with the latest guidelines (Antzelevitch et al., 2017). There was not a clinical suspicion of Brugada Syndrome in any of the patients and for the same reason a genetical testing is also not recommended (Antzelevitch et al., 2017).

Previous studies have reported the prevalence of Type 2 BrP in the general population. Holst et al. (2012) studied 340 healthy subjects and reported an incidence of type 1, 2, and 3 Type 2 BrP on the second ICS, respectively 0, 3.3, and 7.1%. Another work registered similar results in 504 healthy male volunteer subjects. The authors found an incidence of type 1, 2, and 3 Type 2 BrP on 0.8, 2, and 7.5%, respectively (Hunuk et al., 2013). In a population of 491 collegiate athletes a type 2 or 3 Type 2 BrP was seen in 58 (11.8%), and no definitive type 1 was observed (Chung et al., 2014). The much higher incidence of a type 2 or 3 Brugada ECG pattern mostly type 3 in our PVC patients, and its absence in the control group, raises the hypothesis that the two may be associated. Although type 3 ST-segment elevation is no longer regarded as a typical ECG Brugada pattern (Antzelevitch et al., 2017), it was present in a high percentage of our patients and it is not a normal finding either. The term Brugada phenocopy was proposed to describe conditions that induce Brugada-like ECG manifestations in patients without true Brugada syndrome (Baranchuk et al., 2012) and this may be the case.

The second finding in our study was the increase in the percentage of patients with T wave inversion beyond V1 when the ECG was obtained at the level of the second ICS in comparison with the standard ECG position. The diagnosis of ARVC is sometimes difficult, and T wave inversion in V1–V2 is considered a minor criteria (Marcus et al., 2010). If we accept that the T wave inversion at a higher ICS could have a similar value, then the number of ARVC “possible” cases would increase (Marcus et al., 2010). Unlike the ST-segment elevation the presence of T wave inversion was not associated with the presence of LVAs.

The third finding in our study was the presence of LVAs outside the transitional-voltage zone, that was absent in subjects without PVCs. The bipolar voltage above the pulmonary valve is typically less than 0.5 mV or even less than 0.1 mV (Furushima et al., 2012) due to the absence or scarcity of myocardium at that level. The voltage progressively increases as the catheter is withdrawn to the RVOT. The area immediately below the pulmonary valve displays a voltage between 0.5 and 1.5 mV and is described as the transitional-voltage zone (Yamashina et al., 2009). The length of this area is variable and according to the authors, longer in patients with malignant arrhythmias than in those with a benign course. In our study the LVAs were outside the transitional-voltage zone, into the RVOT body. Their presence was not significantly different in patients with a RVOT or LVOT origin. Probably, these results are due to the small number of patients with PVCs from LVOT. However, we cannot rule out the possibility that the PVCs from any of the outflow tracts represent the same disease with different manifestations.

The presence of LVA in patients with idiopathic PVCs is not a recent finding. In fact, a high number of previous studies have already demonstrated this finding, either with conventional ablation catheters (Yamashina et al., 2009; Furushima et al., 2012; Parreira et al., 2019a, b) or recently, with the use of a multipolar catheter to obtain a high−density endocardial voltage mapping (Letsas et al., 2019). The bipolar voltage depends amongst other things on the recording electrode size and the interelectrode spacing. One may argue that with high density/high resolution mapping the results could be different. We found the presence of LVA outside the transitional-voltage zone in 28 out of 45 patients (62%). Letsas et al. (2019) mapped the RVOT of patients with idiopathic PVCs using a multipolar catheter with 2 mm electrodes for high density mapping (mean number of sampled points 1096.6 ± 322.3), and identified at least two low bipolar voltage areas less than 1 mV in 39 out of 44 patients (88%), a higher percentage than ours. These results prove that our high prevalence of LVAs is surely not the result of the lack of multipolar catheters. Those authors used an ablation strategy aiming at these LVAs in patients with low PVC burden as previously proposed by other group (Wang et al., 2016) with good success rates. The presence of LVAs did not match the results of the CMR in any of the studies. Detection of fibrosis using LGE has been well validated in ischemic cardiomyopathy and post-myocardial infarction, with an excellent agreement between CMR findings and the voltage map (Torri et al., 2019). However, that is not true for non-ischemic myocardiopathy. Myocardial fibrosis is a common final pathway in chronic myocardial disease but the type of interstitial fibrosis that occurs in the initial phases of non-ischemic myocardiopathy or ARVC is not reliably detected with LGE, and other techniques are being investigated (Puntmann et al., 2016; Bing and Dweck, 2019). On the other hand, we must not forget the true meaning of LVAs. In fact, low bipolar voltage is not synonyms of fibrosis. Bipolar voltage amplitude is influenced by many variables independently of the presence of fibrosis (Anter and Josephson, 2016). Boulos et al. (2005) studied the voltage map in patients with ARVC, normal subjects and idiopathic PVCs. They found a regional difference in the bipolar voltage throughout the right ventricle, and the RVOT displayed the lowest voltage (Boulos et al., 2005). However, it was well above the 1.5 mV cut-off value (mean 2.6 ± 0.4 mV) in normal subjects and significantly higher than the bipolar voltage in the dysplastic regions of patients with ARVC (0.60 ± 0.06 mV). So, independently of the points discussed above, the presence of LVAs in the middle of normal voltage areas, must be considered an abnormal finding and hopefully a target for ablation as anticipated by the high percentage of cases in whom the site of origin of the arrhythmia was in the LVA, respectively in 18 out of 28 patients (64%) of cases.

The last finding was the association of the Type 2 BrP at higher V1 with the presence of LVAs in patients with apparently normal hearts. We have previously reported this finding with a smaller number of patients, and some limitations namely, the absence of a control group and the fact that CMR was not performed in all patients (Parreira et al., 2019a). The present study confirmed those preliminary results and we were able to demonstrate that the Type 2 BrP in the second ICS was a predictor of LVAs. The remarkably high odds ratio and wide CI is due to an extremely low prevalence of LVA in the absence of Type 2 BrP (one patient) and extremely low prevalence of absent LVAs in patients with Type 2 BrP (two patients). Presence of fibrosis as well as reduced connexin-43 signal was described in the RVOT of autopsies of patients with Brugada syndrome. The authors find therefore plausible that Brugada syndrome may reflect a generalized disease of myocardial of the RVOT predisposing it to fibrosis (Nademanee et al., 2015). The role of fibrosis in Brugada syndrome is uncertain, and the clinical phenotype concomitant with cardiac fibrosis remains a matter of ongoing scientific investigation (Brugada et al., 2018). Recently, the presence of LVAs has been reported in the endocardium of the RVOT of patients with Brugada ECG pattern (Letsas et al., 2018). The authors studied 10 asymptomatic patients with spontaneous type 1 Brugada pattern using high density mapping and found abnormal unipolar and bipolar electrograms displaying areas of low voltage despite normal CMR. None of our patients display a type 1 Brugada pattern but the electrocardiogram performed in a higher position was not normal either. Thus, we may speculate that in RVOT arrhythmias the presence of LVAs may be a marker of a very early stage of disease, not detected by current imaging techniques.

Low voltage areas may be pointed as a possible target for ablation and Type 2 BrP may be used as a non-invasive marker.

This study has some limitations. First, two different mapping systems were used to obtain the voltage map, and patients in the control group were mostly mapped with Carto and Stereotaxis. Nevertheless, the association between the presence of a Type 2 BrP and LVAs was proved with both systems. Secondly, we only considered patients that had a map of the RVOT done, so some of the patients with PVCs that went directly to LVOT mapping were excluded, leaving a small number of patients with the site of origin in the LVOT. A high-density voltage mapping was not performed. The median number of sampled points was 142 (98–300), which could hardly be considered insufficient for such a small area as the RVOT, and as proved by the similar results obtained with high-density mapping (Letsas et al., 2019).

We did not perform angiography to assess the level of the pulmonary valve. However, the LVAs that were analyzed in this study, were outside the transitional-voltage zone. The true level of the pulmonary valve is irrelevant for the interpretation of the results. Regarding the pattern of ST elevation observed in our patients, despite being abnormal, is not considered as a diagnostic Brugada ECG pattern according to the latest guidelines. However, we did not expect our patients to have Brugada Syndrome and for this reason we did not pursue further investigation.

Finally, patients did not repeat the ECG in the second ICS to evaluate if Type 2 BrP persisted after successful PVC ablation.

## Conclusion

In conclusion, LVAs outside the transitional-voltage zone were frequently present in the RVOT of patients with idiopathic PVCs from the outflow tract. Those were absent in controls and could be unmasked by the presence of Type 2 BrP in high right precordial leads. The site of origin of the PVCs were within the LVA in a high percentage of cases. Low voltage areas may be a potential target for PVC ablation and Type 2 BrP is an accurate non-invasive marker of LVAs.

## Data Availability Statement

The raw data supporting the conclusions of this article will be made available by the authors, without undue reservation.

## Ethics Statement

The studies involving human participants were reviewed and approved by the Hospital da Luz Lisboa and Hospital Center of Setúbal. The patients/participants provided their written informed consent to participate in this study.

## Author Contributions

LP contributed to the conceptualization, methodology, and writing. LP, RM, PC, DM, JF, PAm, MF, and AF contributed to the investigation. RM, DM, FC, DC, RC, and PAd contributed to the reviewing. All authors contributed to the article and approved the submitted version.

## Conflict of Interest

The authors declare that the research was conducted in the absence of any commercial or financial relationships that could be construed as a potential conflict of interest.
